# MRI Findings for Frozen Shoulder Evaluation: Is the Thickness of the Coracohumeral Ligament a Valuable Diagnostic Tool?

**DOI:** 10.1371/journal.pone.0028704

**Published:** 2011-12-07

**Authors:** Jin-qing Li, Kang-lai Tang, Jian Wang, Qi-yu Li, Hao-tong Xu, Hui-feng Yang, Li-wen Tan, Kai-jun Liu, Shao-xiang Zhang

**Affiliations:** 1 Department of Anatomy, College of Basic Medical Science, Third Military Medical University, Chongqing, People's Republic of China; 2 Department of Orthopaedic Surgery, Southwest Hospital, Third Military Medical University, Chongqing, People's Republic of China; 3 Department of Radiology, Southwest Hospital, The Third Military Medical University, Chongqing, People's Republic of China; Marienhospital Herne - University of Bochum, Germany

## Abstract

**Background:**

Recent studies have demonstrated that the coracohumeral ligament (CHL) is shortened and thickened in a frozen shoulder. We analyzed the rate in CHL visualization between patients with frozen shoulder and normal volunteers using Magnetic Resonance Imaging (MRI) to determine the CHL thickness in the patients with a frozen shoulder.

**Methods and Findings:**

There were 72 shoulder joints in 72 patients (50 femles and 22 males with a mean age of 53.5 years) with clinical evidence and MR imaging evidence of frozen shoulder. These were prospectively analyzed to identify and measure the maximum thickness of the CHL. The control group, which included 120 shoulder joints in 60 normal volunteer individuals (30 females and 30 males with a mean age of 50.5 years) was also referred for MR imaging. A chi-square test was used to analyze the data of the rate of CHL visualization between the patients with frozen shoulder and the control group. A two-way ANOVA was used to analyze the mean maximal thickness of CHL. The CHL was visualized in 110 out of 120 shoulders in the control group (91.7%), and in 57 out of 72 shoulders for the frozen shoulder group (79.2%), there was significant difference, using a chi-square test (*P*<0.05). The CHL was not visualized in 10 out of 120 shoulders in the control group (8.3%), and 15 out of 72 shoulders in the frozen shoulder group (20.8%), there was a significant difference (*P*<0.05). The CHL thickness (3.99±1.68 mm) in the patients with frozen shoulder was significantly greater than that thickness (3.08±1.32 mm) in the control group, using a two-way ANOVA (*P*<0.001). The CHL thickness (3.52±1.52 mm, n = 97) in the female shoulders was no significantly greater than that thickness (3.22±1.49 mm, n = 70) in the male shoulders, using a two-way ANOVA (*P*>0.05).

**Conclusions:**

MR Imaging is a satisfactory method for CHL depiction, and a thickened CHL is highly suggestive of frozen shoulder.

## Introduction

Frozen shoulder, also known as adhesive capsulitis, is a common condition involving scapulohumeral pain and loss of motion. Our understanding the definition and diagnosis of this disease has slowly progressed. In 1934, Codman described the diagnosis of “frozen shoulder” as a condition characterized by pain and reduced range of motion in the affected shoulder [1]. In 1945, Neviaser used the term “adhesive capsulitis” to describe the findings of chronic inflammation and fibrosis of the joint capsule [2]. But we poorly grasp of the etiology and pathophysiology of frozen shoulder, and frozen shoulder is a poorly defined entity today. The current consensus regarding a definition of frozen shoulder comes from the American Shoulder and Elbow Surgeons and states it is “a condition of uncertain etiology characterized by significant restriction of both active and passive shoulder motion that occurs in the absence of a known intrinsic shoulder disorder.” [3], [4].

Frozen shoulder most commonly affects women between 40 and 60 and is usually diagnosed on the basis of clinical findings and not radiographic findings. However, frozen shoulder can mimic other causes of shoulder pain and stiffness, including calcific tendonitis, bicipital tenosynovitis, glenohumeral and acromioclavicular arthritis, tears of the rotator cuff and shoulder tumors [5]. Accurate diagnosis is essential because of dissimilar treatment approaches for these separate entities.

Recent studies have shown that Magnetic Resonance Imaging (MRI) can provide reliable imaging indicators of frozen shoulder. Potentially useful MR findings in frozen shoulder include thickening of the CHL, thickening of the joint capsule in the rotator cuff interval, and obliteration of the fat triangle under the coracoid process [6]. On MR images obtained after an intravenous gadolinium injection, enhancement of the joint capsule and synovial membrane and enhanced fibrovascular tissue in the rotator cuff interval may be helpful in the identification of frozen shoulder [7]–[9]. Among the numerous MRI findings in frozen shoulder, can the thickness of the CHL then be really taken as one of the most characteristic MR findings for the diagnosis of frozen shoulder?

Some studies have demonstrated that the coracohumeral ligament (CHL) is shortened and thickened in frozen shoulder, thus limiting external rotation. Histological analysis has shown that the limitation of external rotation is due to fibroblastic proliferation within the CHL. Such histological changes are very similar to those found in Dupuytren's superficial fibromatosis [10]–[14]. Mengiardi et al also found that patients with frozen shoulder had a significantly thickened CHL using MR arthrography [6]. To our knowledge, only few studies have answered the question regarding the rate of CHL visualization between patients with frozen shoulder and normal volunteers using routine MRI. In view of the fact that MRI can demonstrate the CHL and this ligament is thickened in frozen shoulder, the purpose of this study is to analyze the rate of CHL visualization between patients with diagnosed frozen shoulder and normal volunteers via use of an MRI and determine the CHL thickness in the patients with frozen shoulder and compare it with that found in the normal volunteers.

## Methods

### Study Population

A total of 72 shoulder joints in 72 patients with clinical evidence of frozen shoulder were prospectively referred by several upper limb orthopedic surgeons for MR imaging. These included 50 females and 22 males with a mean age of 53.5 years (age range 33–72 years). All these patients reported an insidious onset of shoulder pain and dysfunction, ranging from 15 weeks to 18 months in duration (mean of 9.1 months). Their initial diagnosis was made on the basis of history, clinical findings, and routine MRI findings. Clinical criteria for the condition include pain and stiffness for more than fifteen weeks, increasing in nature and most severe at rest with restriction of passive motion greater than 30° for two or more planes of movement. Exclusion criteria included previous trauma, previous shoulder surgery, tumors, rotator cuff tearing, presence of calcium deposit on x-ray evaluation, rheumatoid arthritis, osteoarthritis, cervical spine disease, diabetes mellitus, thyroid disease, heart disease, pulmonary disease, or hemiplegia. All patients had undergone medical treatment that included anti-inflammatory medication, physiotherapy, both of them, and followed up for 24 months. No patients were lost to follow-up.

The control group included 120 shoulder joints in 60 normal volunteer individuals (30 females and 30 males with a mean age of 50.5 years) also referred for MR imaging. Exclusion criteria included rheumatoid arthritis, previous shoulder surgery, previous trauma, and/or abnormal radiographs.

This study was approved by our Institutional Review Boards (IRBs) for Third Military Medical University. All subjects were enrolled with signed written informed consent in accordance with the regulations of both IRBs.

### The MR Imaging Protocol

MR Imaging was performed using a 1.5-T system (Avanto; Siemens Medical Solutions, Germany ). A phased-array surface coil was centered over the glenohumeral joint and strapped in place. The arm position was standardized, with the thumb pointing upward in a neutral position. T1-weighted spin-echo images were obtained in the transverse plane (624/11 [repetition time msec/echo time msec], 3-mm section thickness, 0.3-mm intersection gap, 180×180-mm field of view, and 512×512 matrix size). In the sagittal oblique plane, parallel to the glenohumeral joint (550/15, 3-mm section thickness, 0.3-mm intersection gap, 180×180-mm field of view, 512×512 matrix size), Fat-suppressed proton-density weighted spin-echo images were obtained in the coronal oblique plane, paralleling the long axis of the supraspinatus tendon (3000/34, 3-mm section thickness, 0.3-mm intersection gap, 180×180-mm field of view, and 512×512 matrix size), and obtained for the transverse and sagittal oblique planes.

### Analysis of the MR Images

The thickest portion of the CHL was measured on sagittal T1-weighted spin-echo oblique images by a fellow in musculoskeletal radiology, who was blinded to the diagnosis. The joint capsule in the rotator cuff interval and in the axillary recess was analyzed via consensus by two staff radiologists with 8 years and 20 years of experience in musculoskeletal radiology.

### Statistical Analysis

Statistical analysis was completed using the SPSS software program (version 13.0 for Windows; SPSS, Chicago, IL). A chi-square test was used to analyze the data for the rate in CHL visualization between the patients with frozen shoulder and the control group. A two-way ANOVA was used to analyze the maximum thickness of the CHL. The results of the CHL thickness were then expressed as mean ±SD for each group. Two-tailed hypothesis tests were used and local statistical significance was assumed to be *P*<0.05 for all parameters.

## Results

**Figure 1:**
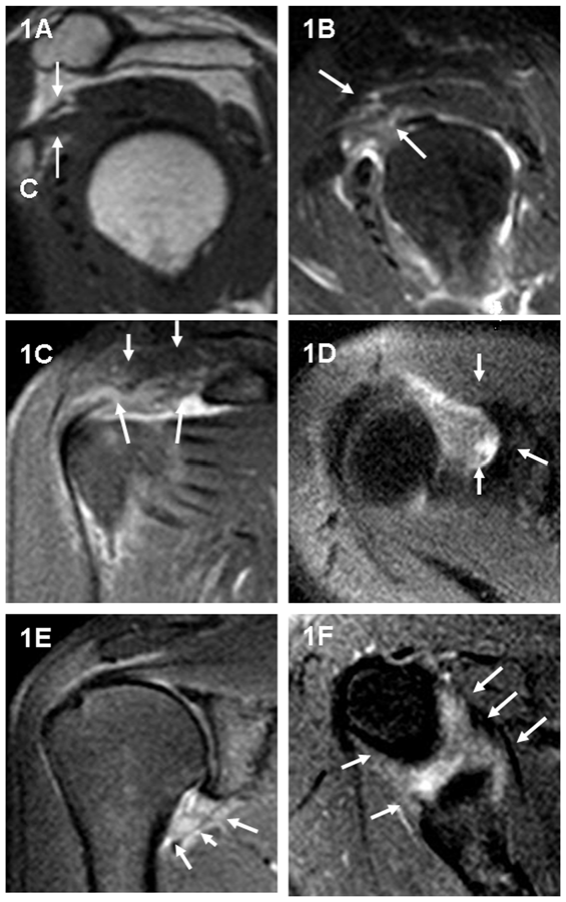
A 56-year-old female patient with clinical evidence of a right frozen shoulder. Sagittal oblique T1-weighted image (TR/TE = 550 ms/15 ms) (1A) shows thickened CHL (arrows). C = inferior margin for the coracoid process. Sagittal oblique (1B), oblique coronal (1C), and transverse (1D) fat-suppressed, proton density weighted, spin-echo image (TR/TE = 3000 ms/34 ms) show high-signal intensity soft tissue in the rotator cuff interval for the same patient (arrows). Coronal oblique (1E) and transverse (1F) fat-suppressed, proton density, weighted spin-echo image (TR/TE = 3000 ms/34 ms) demonstrate a thickened inferior glenohumeral ligament (axillary recess) for the same patient (arrows).

Fat-suppressed, proton-density weighted, spin-echo images of the shoulder demonstrated high-signal intensity soft tissue in the rotator cuff interval seen on the sagittal oblique, coronal oblique and transverse images, a thickened inferior glenohumeral ligament (axillary recess) seen on the coronal oblique and transverse images, a low-signal intensity thickened CHL seen on the sagittal oblique T1-weighted image (Figure 1). These findings were compatible with frozen shoulder.

**Figure 2:**
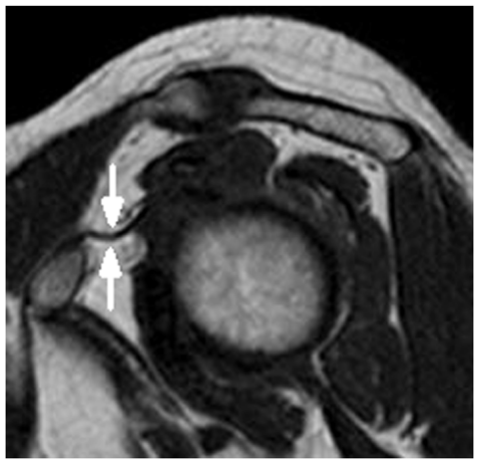
A 49-year-old male normal volunteer. Sagittal oblique, T1-weighted image (TR/TE = 550 ms/15 ms) demonstrates a normal CHL showing homogeneous , low signal intensity (arrows).

**Figure 3:**
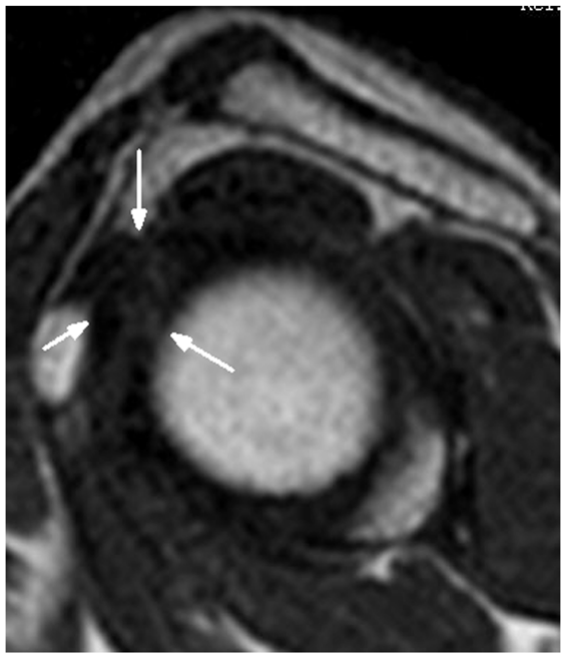
A 54-year-old female normal volunteer. Sagittal oblique, T1-weighted image (TR/TE = 550 ms/15 ms) shows that the distinct fatty tissue surrounding the CHL had disappeared , and the CHL could not be measured (arrows).

**Figure 4:**
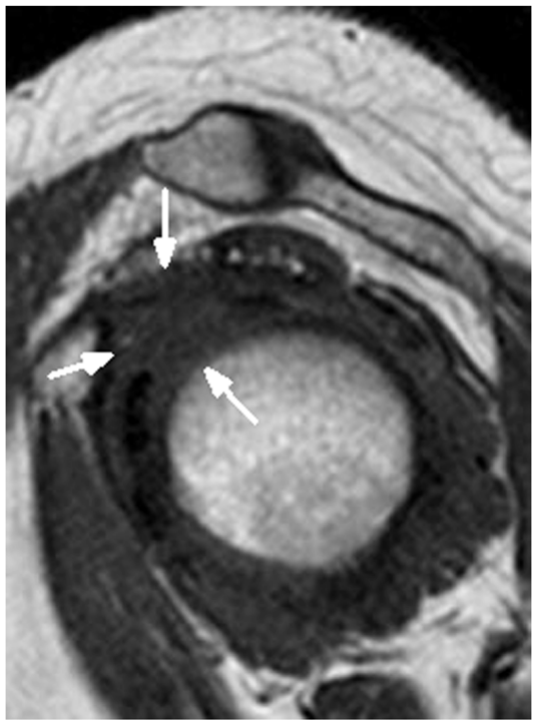
A 57-year-old male patient with clinical evidence of right frozen shoulder. Sagittal oblique, T1-weighted image (TR/TE = 550 ms/15 ms) shows the complete obliteration of subcoracoid fat triangle and distinct fatty tissue surrounding the CHL as having disappeared, and the CHL cannot be measured (arrows).

In contrast, these findings were not seen in the control group (Figure 2). However, a percentage of shoulders of both the control group and the frozen shoulder group demonstrated that the complete obliteration of subcoracoid fat triangle and distinct fatty tissue surrounding the CHL had disappeared, so the CHL could not be measured for the sagittal oblique images (see Figure 3 and Figure 4).

A comparison of the rate of CHL visualization in the patients with frozen shoulder versus the control group was given in Table 1. The CHL was visualized in 110 out of 120 shoulders in the control group (91.7%), and 57 out of 72 shoulders in the frozen shoulder group (79.2%), and there was significant difference, using the *x* ^2^ test (*P* = 0.013). In the control group, the CHL visualization rate (95%) in the female shoulders was compared with that rate (88.3%) in the male shoulders, there was no significant difference (*P* = 0.186). In the frozen shoulder group, the CHL visualization rate (80%) in the female shoulders was compared with that rate (77.3%)in the male shoulders, there was also no significant difference (*P* = 0.793). When the rate of CHL visualization for the female shoulders in the control group (95%) was compared with that rate in the female frozen shoulders (80%), there was a significant difference (*P* = 0.015). However, when the rate of CHL visualization for the male shoulders in the control group (88.3%) was compared with that rate in the male frozen shoulders (77.3%), there was no significant difference (*P* = 0.209). The CHL was not visualized in 10 out of 120 shoulders in the control group (8.3%), and 15 out of 72 shoulders in the frozen shoulder group (20.8%) (*P*<0.05).

**Table 1:** Comparison of the Rate of CHL Visualization in the Patients with Frozen Shoulder versus Control Group.

	Patients with Frozen Shoulder	Control Group	
	(n = n1+n2 = 72)	(n = n1+n2 = 120)	
Gender	n(%)	n(%)	*P*-value*
Female	40(80%)(n1 = 50)	57(95%)(n1 = 60)	0.015
Male	17(77.3%)(n2 = 22)	53(88.3%)(n2 = 60)	0.209
Total	57(79.2%)(n = 72)	110(91.7%)(n = 120)	0.013

*Compared by using the Chi-square test.

A comparison of the CHL thickness (mean ± SD) in the patients with frozen shoulder versus the control group was as follows. The CHL thickness (3.99±1.68 mm) in the patients with frozen shoulder was significantly greater than that thickness (3.08±1.32 mm) for the control group, using an a two-way ANOVA (*P*<0.001). The CHL thickness (3.52±1.52 mm, n = 97) in the female shoulders was no significantly greater than that thickness (3.22±1.49 mm, n = 70) in the male shoulders, using a two-way ANOVA (*P* = 0.533). In the frozen shoulder group, the CHL thickness in the female frozen shoulder was 4.21±1.78 mm (n = 40), and that thickness in the male frozen shoulder was 3.47±1.34 mm (n = 17). In the control group, the CHL thickness in female shoulders was 3.03±1.08 mm (n = 57) while it was 3.14±1.54 mm (n = 53) in male shoulders.

## Discussion

Although the precise pathogenesis of frozen shoulder is not known, the CHL is shortened and thickened in frozen shoulder, and a thickened CHL is considered to be one of the most characteristic manifestations of frozen shoulder [10]–[14]. The CHL appears flat with a homogeneous, low-signal intensity linear band surrounded by hyper-intensity fat tissue in T1-weighted spin-echo images (Figure 2). The CHL is always well identified in the mid-portion of the rotator cuff interval and is visualized on all planes, but as the sagittal images are the most useful for analysis of this structure by MRI [15], the thickest portion of the CHL was measured on sagittal, T1-weighted, spin-echo oblique images in our study (see Figure 1 and Figure 2).

Only few articles pointed out the rate of the visualized CHL. Our study indicated that there is significant difference in the rate of CHL visualization between patients with frozen shoulder (79.2%) and the control group (91.7%) (*P*<0.05). In the control group, the CHL was visualized in 110 out of 120 shoulders in the control group (91.7%), near to Neer et al's findings that indicating the rate of the visualized CHL in anatomic specimens of the shoulder in cadaveric studies was about 93.7% (59/63) [13], higher than Chung et al's report stating that the CHL was seen in only 60% of the specimens by the MRI [16], and higher than the results of Homsi et al where 76.0% (92/121) of the CHL was visualized in the asymptomatic group, 63.0% (227/360) of the CHL in the painful shoulder group, and 66.9% (334/498) in shoulders overall using Ultrasound [17]. Neer et al reported that the CHL was not visualized in 4 out of 63 (6.7%) anatomic specimens of the shoulder because the CHL was absent or vestigial. Homsi et al showed that the inherent difficulty in positioning and moving painful shoulders can be one of the reasons for a lower Ultrasound visualization rate of the CHL in the symptomatic group (63%) than in the asymptomatic group (76%). However, Homsi et al did not explain the reasons that 24.0% (29/121) of the CHL was not visualized in the asymptomatic group, using Ultrasound. The CHL measurement depended on the hyper-intensity fat surrounding the CHL in the rotator cuff interval distinguishing from the hypo-intensity CHL. We can not explain the reasons that the fat surrounding the CHL in the rotator cuff interval disappeared in normal volunteer shoulders. In the control group, we found that fat-suppressed, proton density weighted, spin-echo images did not show high-signal intensity soft tissue in the rotator cuff interval in normal volunteer shoulders. We suspected that the fat surrounding the CHL in the rotator cuff interval might be absent in a percentage of normal shoulders, which needed to be further confirmed in the future study.

In the frozen shoulder group, the CHL was visualized in 57 out of 72 shoulders in the frozen shoulder group (79.2%), lower than the results by Homsi et al of 88.2% (15/17) in the adhesive capsulitis group [17]. With adhesive capsulitis, the higher Ultrasound visualization rate of the CHL (88.2%) can be explained by the greater thickness and conspicuity of the CHL. When we compared the CHL visualization rate for the female shoulders between the control group (95%) and the frozen shoulder group (80%), there was a significant difference (*P*<0.05). This result may relate to the fact that frozen shoulder most commonly affected female patients in our study (50 females *vs.* 22 males). The CHL was not visualized in the frozen shoulder group (20.8%) because the distinct fatty tissue surrounding the CHL disappeared, and the thickness of the CHL could not be measured on sagittal T1-weighted spin-echo oblique images (see Figure 3 and Figure 4). But, fat-suppressed, proton density weighted, spin-echo images showed high-signal intensity soft tissue in the rotator cuff interval which was useful for diagnosing the frozen shoulders. We suspected that the nonfatty soft tissue infiltration in the rotator cuff interval fat for a percentage of frozen shoulders, which caused to the CHL thickness not to be measured. Hence, the CHL visualization rate in female shoulders might be suggestive of frozen shoulder, especially lower than 80%. Of course, more studies are needed for clinical validation of CHL visualization rate diagnosing the frozen shoulder in the future. In addition, That the thickness of the CHL could not be measured may be explained by the lack of MR arthrography as the report of Mengiardi et al indicated [6]. MR arthrography may improve the rate of CHL visualization of the shoulder because shoulder MR arthrography has been shown to be significantly more sensitive for certain shoulder pathologies including partial-thickness articular surface supraspinatus tears, anterior labral tears, and SLAP tears [18]. In the future study, shoulder MR arthrography might be useful to evaluate the CHL visualization. However, our study leads one to suspect that the complete obliteration of the subcoracoid fat triangle was specific for the diagnosis of frozen shoulder as Mengiardi et al did report [6]. That is to say, the complete obliteration of the subcoracoid fat triangle was useful, but not specific for the diagnosis of frozen shoulder. To sum up, our study indicates that MRI is a quite satisfactory method for depicting the CHL because the higher MRI visualization rate for the CHL in the control group (91.7%) is similar to that for anatomic specimens of the shoulder in cadaveric studies (93.7%), and because the visualization rate for the CHL was near to 80% in the frozen shoulder group. In addition, the CHL visualization rate in female shoulders might be suggestive of frozen shoulder, especially lower than 80%.

The thickness of the CHL is a useful criterion to use for diagnosis of frozen shoulder. Our study indicated that patients with frozen shoulder had a significantly thickened CHL (3.99±1.68 mm in frozen shoulders *vs.* 3.08±1.32 mm in controls), near to the CHL thickness (4.1 mm *vs.* 2.7 mm in controls) found by Mengiardi et al [6]. In addition, the CHL thickness (3.52±1.52 mm, n = 97) in the female shoulders was no significantly greater than that thickness (3.22±1.49 mm, n = 70) in the male shoulders, using a two-way ANOVA (*P*>0.05). That is to say, the CHL thickness was a significant difference between patient shoulders and control group shoulders(*P*<0.001), and no significant difference between the female shoulders and the male shoulders (*P*>0.05). The CHL thickness in our study was greater than that found by Homsi et al in which 3 mm of adhesive capsulitis was shown, 1.34 mm in the asymptomatic shoulders and 1.39 mm in painful shoulders with ultrasound [17]. Thickness measurements with Ultrasound, as reported by Homsi et al, were performed on a stretched CHL with the shoulder in a neutral position and the forearm extended. This positioning might explain the smaller average values compared to the results found in our study when the thumb was pointed upward in a neutral position.

We acknowledge several limitations for our study. First, our study lacked MR arthrography diagnosis and surgical confirmation of frozen shoulder. Second, our study was done with just one institute and was based on a small sample, factors that might cause a certain selection bias in terms of the patients with frozen shoulder. A large-scale, multi-centered, randomized, prospective study should be undertaken in the future to further study the issue that a thickened CHL is highly suggestive of frozen shoulder. Third, our study included a lack of interobserver and intraobserver correlation to CHL measurement.

In conclusion, MR Imaging is a satisfactory method for CHL depiction and a thickened CHL is highly suggestive of frozen shoulder, perhaps taken as one of the most characteristic MR findings for frozen shoulder.
